# Laboratory and dialysis characteristics in hemodialysis patients suffering from chronic itch - results from a representative cross-sectional study

**DOI:** 10.1186/s12882-015-0177-3

**Published:** 2015-11-04

**Authors:** Elke Weisshaar, Melanie Weiss, Jutta Passlick-Deetjen, Ulrich Tschulena, Klaudia Maleki, Thomas Mettang

**Affiliations:** Department of Clinical Social Medicine, Occupational and Environmental Dermatology, University Hospital, Ruprecht Karls University, Thibautstrasse 3, 69115 Heidelberg, Germany; Department of Nephrology, University Hospital, Duesseldorf, Germany; Fresenius Medical Care Deutschland GmbH, Bad Homburg, Germany; Department of Nephrology, DKD Helios Clinic, Wiesbaden, Germany

**Keywords:** Hemodialysis, Itch, Uremic pruritus, Laboratory values, Pruritus

## Abstract

**Background:**

A representative cross-sectional study showed that chronic itch (lasting for a minimum of 6 weeks) affects 25.2 % (point prevalence) of hemodialysis (HD) patients. Pathophysiology and etiology of chronic itch (CI) in HD are still unclear.

**Methods:**

We investigated 860 HD patients from a representative randomly selected cluster-sample considering the regional distributions of dialysis units in GermanyThe current analyses report comorbidities, laboratory values and dialysis characteristics of HD patients in relation to CI.

**Results:**

Diabetes was the only comorbidity that was associated with the occurrence of itch but interestingly with less CI. Except for creatinine, phosphorus, and parathormone, there were no significant associations between the occurrence and characteristics of CI and any laboratory value. Kt/V was not associated with the presence of CI. Patients dialyzed with polyarylethersulfone-membrane showed significantly more CI in all prevalence estimates and those dialyzed with polysulfone-membrane were significantly less affected by CI.

**Conclusions:**

Long-term follow-up studies will show if the type of dialysis membrane influences the development of CI in HD patients. It is most likely that several factors e.g. elevated parathormone, origin of end stage renal disease (ESRD), type of dialysis membrane, and a neuropathic component all contribute to the occurrence of CI in HD patients. Future research should consider a multifactorial origin of itch in HD.

## Background

GEHIS (German Epidemiological Hemodialysis Itch Study) is a cross-sectional study that investigated 860 hemodialysis (HD) patients from a randomly selected cluster-sample (considering the regional distribution of dialysis units in Germany) aiming to identify different prevalence estimates of chronic itch [1]. According to the international classification, chronic itch (CI) is defined as itch lasting for 6 weeks and longer [2]. This classification had never been applied in a cohort of hemodialysis patients except for GEHIS [1]. Our first analyses revealed that CI affects 25.2 % (point prevalence) of HD patients. 35.2 % reported to have suffered from chronic itch (CI) at least once in their life (lifetime prevalence) and 27.2 % reported CI within the past 12 months [1]. CI was significantly less prevalent in patients with an etiology of secondary glomerulonephritis. There was a significant association of the time since HD treatment started and the occurrence of CI. General health status and health-related quality of life (HRQOL) were significantly more impaired in those suffering from CI [1]. All this demonstrates that CI in HD patients is a frequent, long-lasting burden significantly impairing patient’s health.

The role of comorbidities in the occurrence of itch in HD patients is unclear and has so far only been investigated for selected comorbidities [3–5]. Single studies investigated some abnormalities of laboratory parameters in HD patients suffering from itch e.g. parathormone (PTH), calcium, phosphorus, but the role of them in the occurrence and the intensity of CI in HD patients is still unclear [3, 5–9]. So far, there is no systematic investigation on dialyzer membrane use and the development of CI. Smaller studies hint to conflicting results regarding the role of dialyzer membranes [10–12]. The current analyses investigated possible associations of medical (etiology of end-stage renal disease (ESRD), comorbidities), laboratory and dialysis characteristics (time, duration, dialyzer membrane, anticoagulation) with the occurrence and characteristics of CI.

## Methods

The study (GEHIS) was established as a prospective observational prevalence study in 2012 and was conducted between May and September 2013. Eligible patients were diagnosed with ESRD and were undergoing chronic hemodialysis (HD) treatment. The study was established according to the guidelines of good clinical practice and conducted in full accordance with the protocols of the World Medical Association’s Declaration of Helsinki. Ethical approval was obtained from the University of Heidelberg, Germany (No. S-648/2012). Patients gave their written informed consent. All results were reported in line with the “Strengthening the Reporting of Observational Studies in Epidemiology (STROBE)” recommendations [13]. A detailed description of the study, its design and the study instruments are provided elsewhere [1].

The current analyses are based on a data set obtained in every patient including the etiology of ESRD (primary glomerulonephritis, secondary glomerulonephritis including systemic diseases such as diabetes mellitus, pyelonephritis/interstitial nephritis, hypertensive nephritis, congenital renal disease, unknown origin, others) and sociodemographic data (sex, age, occupational status, education, marital status, ethnic origin) 1]. Itching was assessed asking for current CI, CI within the last 12 months and CI ever in life as well as characteristics of CI [1]. Severity of itch was measured using a visual analogue scale (VAS) ranging from 0 (no itching) to 10 (maximum imaginable itching) which proved to be a valuable method of itch assessment [14].

Routine laboratory investigations were usually performed every four weeks (except for PTH). Laboratory values closest to the date of investigation were transferred from the laboratory chart: leukocytes (G/l), haemoglobin (Hb) (g/dl), haematocrit (Hct) (%), platelets (G/l), albumin (g/dl), creatinine (mg/dl), urea (mg/dl), glucose (mg/dl), sodium (mmol/l), potassium (mmol/l), calcium (mmol/l), phosphate (mmol/l), C-reactive-protein (CRP) (mg/dl), iPTH (ng/L). PTH values older than 6 month were not considered. Different assays have been used for PTH measurement with different reference values. This was considered in our analyses. The following laboratory values were also collected if available in the chart: magnesium, alkaline phosphatase, transaminases, gamma-GT, serum albumin, IgE, eosinophils, hepatitis and HIV-status.

An adapted version of the Charlson Comorbidity Index was used to assess comorbidities in patients with ESRD [15, 16]. Additional comorbidities of the patients if present were assessed according to the patients’ charts.

Kt/V was extracted from the laboratory files selecting the last available value within the last 4 months. Calculation of Kt/V was performed in accordance to the German quality assurance regulation (single pool) using the Daugirdas approximation by each centre [17]. The following dialysis parameters were assessed: start and weekly duration of HD treatment, mean dialysate temperature, type of dialysis membrane (see Table 4), disposable sterilisation method (beta radiation, gamma radiation, ethylene oxide and steam), type of membrane flux (low-flux, high-flux), type of heparin (unfractionated heparin, low molecular heparin, other anticoagulants) used during HD and pre-flushing.

### Statistical analyses

For data management, a Microsoft Access 2003 database was used. Data entry was conducted twice by two independent persons. To maximize data quality, all observed random or potentially systematic inconsistencies within resulting data were solved. Statistical analyses were performed using *SPSS* (version 20) for Windows. Nominal and ordinal data were analyzed by computing absolute (*n*) and relative frequencies (%), respectively, and 95 % confidence intervals were computed. First chi^2^ statistics were used to identify variables that were significantly associated with CI in univariate analysis. *P*-values below 0.05 were considered significant. Comparisons between HD patients suffering from CI and HD patients not affected were conducted by independent t-tests/ANOVA for the continuous variables (e.g. laboratory values) and by chi^2^-test for binary distributions. Associations between laboratory findings and the occurrence of CI are reported by Pearson’s correlation coefficients. To deal with the problem of multiple testing when analyzing differences in laboratory values, a Bonferroni correction was conducted, setting the significance cut-off at α/n.

## Results

The study sample has been described in detail elsewhere [1]. 860 HD patients were included into GEHIS, 57.2 % were male. The mean age was 67.2 years (standard deviation (SD) 13.4). At the time of the investigation, 25.2 % (95 %-confidence interval (95 %-CI) 22.4–28.1) suffered from CI (point prevalence, whereby the 12-month prevalence was 27.2 % (95 %-CI 24.1–30.3) and the lifetime prevalence was 35.2 % (95 %-CI 31.9–38.3) [1].

### Comorbidities and chronic itch

Leading comorbidities according to Charlson Comorbidity Index adapted for patients with ESRD were diabetes mellitus in 38.0 % (*n* = 327), congestive heart failure in 24.7 % (*n* = 212) and peripheral arterial occlusive disease in 21.2 % (*n* = 182) (Table 1) [16].

**Table 1:** Comorbidities according to Charlson adapted for patients with ESRD and additional concomitant diseases (*n* = 860)

	Chronic Itch (all prevalence estimates)		Total
	Yes, % (*n*)	No, % (*n*)	(*n* = 860)
	(*n* = 307)	(*n* = 553)	
Comorbidities (according to Charlson)	Relative frequency, % (*n*)	Relative frequency, % (*n*)	Relative frequency, % (*n*)
Myocardial infarction	16.6 (51)	16.8 (93)	16.7 (144)
Congestive heart failure	22.8 (70)	25.7 (142)	24.7 (212)
Peripheral vascular disease	20.0 (62)	21.7 (120)	21.2 (182)
Cerebral vascular disease	12.7 (39)	15.7 (87)	14.7 (126)
Dementia	1.6 (5)	1.8 (10)	1.7 (15)
Chronic lung disease	20.8 (64)	18.6 (103)	19.4 (167)
Rheumatological disease	2.9 (9)	2.9 (16)	2.9 (25)
Peptic ulcer disease	7.8 (24)	8.5 (47)	8.3 (71)
Hemiplegia	1.3 (4)	2.5 (14)	2.1 (18)
Diabetes without complications	30.9 (95)*	42.0 (232)**	38.0 (327)
Diabetes with complications	22.8 (70)*	31.8 (167)**	28.6 (246)
Mild liver disease	5.2 (16)	4.3 (24)	4.7 (40)
Moderate/severe liver disease	2.0 (6)	1.4 (8)	1.6 (14)
Metastatic disease	13.0 (40)	12.1 (67)	12.4 (107)
Leukemia	0.3 (1)	0.2 (1)	0.2 (2)
Lymphoma	3.9 (12)	3.1 (17)	3.4 (29)
Human immunodeficiency virus	-	-	-
Charlson Comorbidity Index, mean ± SD	4.1 ± 4.4	4.4 ± 4.6	4.3 ± 4.5
Charlson score 0	25.7 (79)	23.3 (129)	24.2 (208)
Charlson score 1–2	18.9 (58)	17.5 (97)	18.0 (155)
Charlson score 3–4	19.5 (60)	20.6 (114)	20.0 (174)
Charlson score ≥5	35.8 (110)	38.5 (213)	37.6 (323)
Additionally assessed comorbidities			
Arterial hypertension	87.6 (269)	87.5 (484)	87.6 (753)
Anemia	95.1 (292)	92.9 (514)	93.7 (806)
Hyperparathyroidism	85.0 (261)	83.2 (460)	83.8 (721)
Dyslipidemia	39.7 (122)	41.4 (229)	40.8 (351)
Osteoporosis	6.2 (19)	5.1 (28)	5.5 (47)

*Significantly lower than average at *p* < 0.05

**Significantly higher than average at *p* < 0.05

Evaluating the Charlson comorbidities separately, diabetes mellitus with and without complications was significantly associated with all prevalence estimates of CI. HD patients affected by diabetes mellitus suffered significantly less from CI. Any further significant associations between the prevalence of CI and comorbidities were not identified. Mean score for Charlson comorbidity index adapted for patients with ESRD was 4.3 (SD 4.5) (Table 1). There were no significant associations with the prevalence of CI, neither with the mean score of the Charlson Comorbidity index nor when regarding grouped scores. 87.6 % (*n* = 753) of the HD patients suffered from arterial hypertension, 93.7 % (*n* = 806) from anemia and 83.8 % (*n* = 721) from hyperparathyroidism. Dyslipidemia was observed in 40.8 % (*n* = 351), osteoporosis in 5.5 % (*n* = 47). These comorbidities did not show any significant association with the prevalence of CI (Table 1). None of the investigated patients was HIV positive or had AIDS.

There were no associations of any comorbidities, laboratory values, or type of dialyzer membrane used with the characteristics of itch e.g. localization and severity of CI.

### Laboratory values and chronic itch

All laboratory values are presented in Table 2. 81.9 % of the HD-patients had PTH levels lower than 500 ng/l and in 18.1 % PTH levels were 500 ng/l and higher. There were weak but statistically significant differences in parathormone (PTH), creatinine and phosphorus. These laboratory values were significantly higher in HD patients suffering from CI. There was no correlation between the severity of itch (VAS) and the PTH level. All other laboratory parameters mentioned above were not included in our analyses because not every dialyses unit had documented them and therefore the number of cases was too low (non-routine laboratory parameters).

**Table 2:** Laboratory values (whole population) in patients with and without chronic itch (*n* = 860)

	Current chronic itch (point prevalence)		Total
	Yes	No	(*n* = 860)
Laboratory value, means ± SD^1^	Mean ± SD^1^	Mean ± SD^1^	Mean ± SD^1^
Kt(V)	1.5 ± 0.4	1.5 ± 0.3	1.5 ± 0.3
Urea (mg/dl)	127.6 ± 38.8	132.3 ± 36.3	128.8 ± 38.2
CRP (mg/dl)	1.2 ± 2.1	1.3 ± 1.8	1.2 ± 1.9
Parathormone (ng/l)	376.9 * ± 380.2	289.9 * ± 329.4	311.7 ± 344.6
Hemoglobin (g/dl)	11.4 ± 1.3	11.3 ± 1.2	11.3 ± 1.2
Creatinine (mg/dl)	8.9 * ± 2.9	7.8 * ± 2.6	8.1 ± 2.8
Calcium (mmol/l)	2.2 ± 0.2	2.2 ± 0.2	2.2 ± 0.2
Phosphorus (mmol/l)	1.9 * ± 0.6	1.7 * ± 0.5	1.8 ± 0.5
Potassium (mmol/l)	5.3 ± 0.7	5.2 ± 0.7	5.2 ± 0.7
Sodium (mmol/l)	138.6 ± 3.2	138.6 ± 3.6	138.6 ± 3.5
Alkaline phosphatase (U/l)	104.1 ± 65.6	96.2 ± 78.0	98.1 ± 75.1
Serum albumin (g/dl)	3.9 ± 0.4	3.9 ± 0.5	3.9 ± 0.5

^1^ Standard deviation

* Significantly different at *p* < 0.004 (Bonferroni correction)

### Dialysis characteristics

According to the current quality criteria on HD treatment in Germany, HD patients undergo at least 4 h HD dialysis treatment 3 times weekly and HD treatment aims at a Kt/V of >1.3. The mean dialysis efficacy (Kt/V) was 1.5 (SD 0.3, min 0.5, max 3.1) (Tables 2 and 3). The mean dialysis time per week was 12.9 h (SD 1.3 h) (Table 3). The mean time on HD was 58 months (4.8 years) (SD 56.2 months). HD patients affected by CI (all prevalence estimates) were significantly longer on HD (58 months, SD 56.2 months versus 50.8 SD 45.5 months) [1].

**Table 3:** Dialysis parameters (whole study population) in patients with and without chronic itch (*n* = 860)

	Chronic itch (lifetime prevalence)		Total
	Yes (*n* = 307)	No (*n* = 553)	(*n* = 860)
Dialysis vintage	Mean	Mean	Mean
Start of dialysis treatment, months ± SD^a^	70.4 ± 70.1*	50.8 ± 45.5*	57.5 ± 56.2
Duration of weekly dialysis treatment, hours ± SD	13.0 ± 1.3	12.9 ± 1.3	12.9 ± 1.3
Method of dialysis treatment (*n* = 851)	Relative frequency, % (n)	Relative frequency, % (n)	Relative frequency, % (n)
Hemodialysis	80.1 (246)	81.3 (442)	80.8 (688)
Online hemodiafiltration (post-dilution)	17.3 (53)	17.1 (93)	17.2 (146)
Online hemodiafiltration (pre-dilution)	2.6 (8)	1.7 (9)	2.0 (17)
Dialyzers used (*n* = 850)			
Low-flux	8.8 (27)	12.7 (69)	11.3 (96)
High-flux	91.2 (279)	87.3 (475)	88.7 (754)
Dialyzer membrane (*n* = 848)			
Polysulfone	57.8 (175)**	66.2 (369)	63.3 (544)
Polyethersulfone	10.2 (31)	9.7 (54)	9.9 (85)
Polyarylethersulfone	29.7 (90)***	21.7 (121)	24.5 (211)
Polyacrylonitrile	-	0.2 (1)	0.1 (1)
Polyester-polymer	-	-	
Polymethylmethacrylate	1.7 (5)	0.4 (2)	0.8 (7)
Sterilization technique, blood lines (*n* = 852)			
Beta radiation	37.5 (115)	38.2 (208)	37.9 (323)
Gamma radiation	17.6 (54)	18.5 (101)	18.2 (155)
Ethylene oxide	-	-	-
Steam	45.0 (138)	43.3 (236)	43.9 (374)
Sterilization technique dialyzer (*n* = 852)			
Beta radiation	0.3 (1)	0.6 (3)	0.5 (4)
Gamma radiation	19.2 (59)	20.6 (112)	20.1 (171)
Ethylene oxide		-	-
Steam	80.5 (247)	78.9 (430)	79.5 (677)
Dialysate temperature, mean ± SD (°C) (*n* = 860)	36.4 ± 0.2	36.4 ± 0.3	36.4 ± 0.3

^a^SD: standard deviation

*Significantly different at *p* < 0.05

**Significantly lower than average at *p* < 0.05

***Significantly higher than average at *p* < 0.05

Regarding the methods of dialyzing used, HD treatment was the leading procedure (80.0 %), followed by post-dilution hemodiafiltration (17.2 %) and pre-dilution hemodiafiltration (2.0 %). A significant association of the dialyzing method and the occurrence of CI did not occur. Also differences in dialyzers used (high-flux 88.7 % and low-flux 11.3 %) were not significantly associated with the occurrence of CI. However, the difference in sample size of these two groups may explain why we could not detect significant differences.

Steam was the most commonly used method of sterilization in both blood lines (43.9 %) and dialyzers (79.5 %) (Table 3). In blood lines, the second most frequent method of sterilization was beta radiation (37.9 %), in dialyzers gamma radiation (18.2 %). There were no significant associations between the procedure of sterilization and the occurrence of CI. Regarding the mean temperature of the dialysate (36.4 °C), there were no significant differences in patients affected and not affected by CI.

Anticoagulation was documented for 818 patients. Out of those, unfractionated heparin (UFH) was used for anticoagulation in 81.7 % (*n* = 668) and low molecular weight heparin in 16.5 % (*n* = 135). 1.8 % received another medication for anticoagulation (*n* = 15). There was no significant association of the type of heparin and current CI (point prevalence) but low molecular heparin use was significantly associated with a higher prevalence of CI ever experienced in life. Multiple testing showed that CI was significantly more prevalent (all prevalence estimates) in low molecular heparin use compared to those patients who received another type of anticoagulation (e.g. point prevalence 31.9 % versus 24.0 %). There was no significant association between heparin used and any other factors previously identified e.g. self-reported dry skin, eczema 1].

The following dialyzer membranes were used: polysulfone (63.3 %, *n* = 544), polyarylethersulfone (24.5 %, *n* = 211), polyethersulfone (9.9 %, *n* = 85), polymethylmethacrylat (0.8 %, *n* = 7) and polyacrylonitril (0.1 %, *n* = 1) (Table 4). When looking at the lifetime prevalence of CI, patients dialyzed with a polysulfone membrane were significantly less affected by CI. Patients dialyzed with polyarylethersulfone membrane reported significantly more CI in all prevalence estimates (Table 4). When asking for the severity of “worst itch” experienced according to VAS, patients dialyzed on polysulfone reported significantly higher values on VAS (6.9 in polysulfone membrane vs. 6.0 when using another membrane). There were no other significant associations between the type of membrane and any characteristics of CI.

**Table 4:** Type of dialyzer membrane used in hemodialysis patients with and without chronic itch (*n* = 848)

Dialyzer membrane	Total number of used dialyzer membrane	Chronic itch according to dialyzer membrane, relative frequency (*n*)	
		Yes (12-month prevalence)	No
polysulfone, % (*n*)	63.3 (544)	25.4 (138)	74.6 (406)
polyethersulfone, % (*n*)	9.9 (85)	24.7 (21)	75.3 (64)
polyarylethersulfone, % (*n*)	24.5 (211)	33.6 (71)*	66.4 (140)
polyacrylonitrile, % (*n*)	0.1 (1)	-	100.0 (1)
polyester polymer, % (*n*)		-	-
polymethylmethacrylate, % (*n*)	0.8 (7)	28.6 (2)	71.4 (5)
12-month prevalence		25.2 (215)	

^*^Significantly different at *p* < 0.05

Previous analyses showed that CI was less prevalent in those HD patients with secondary glomerulonephritis as the cause of renal failure [1]. Further analyses grouping all patients according to their aetiology of renal failure did not reveal any significant differences in comorbidities, dialysis characteristics and laboratory parameters.

## Discussion

Itch in ESRD and in HD may be termed uremic itch or “chronic kidney disease-associated itch” and mostly occurs chronically as so-called chronic itch (CI) [18]. It has been shown to be an underestimated, burdensome and therapy-refractory symptom strongly affecting quality of life [1, 19, 20]. GEHIS is the first representative cross-sectional study investigating precise prevalence estimates of CI in HD patients showing that 25.2 % are affected and that 35.2 % ever experienced CI in life 1]. This is also the very first study that used the international classification of CI in hemodialysis patients [2].

The current analyses confirm previous research about the high frequency of comorbidities like congestive heart failure and hypertension in HD patients. Unlike others showing significant differences between patients with and without CI for congestive heart failure and peripheral vascular disease (significantly more frequent in patients with CI), we cannot confirm this. This may be explained by the large numbers of patients investigated in the DOPPS study increasing the likelihood of detecting significant differences [3, 5]. Whereas others found a higher prevalence of itch in HD patients with liver disease the proportion of patients with liver disease was small in our study [4]. In this study from Turkey, which, however is not a representative one, 20 % of all patients investigated suffered either from Hepatitis B or C. As liver diseases can also cause itch, other aetiologies of itch need to be considered in this cohort. A previous study showed itch not to be significantly different between diabetic and non-diabetic HD patients [21] but this result is in contradiction to other studies, which found a higher prevalence in diabetic patients [3, 5, 20]. We found CI in diabetic patients (with and without complications) to be significantly less prevalent which is an unexpected and striking finding. However, it was shown that neuropathy was significantly more common in HD patients with itch [21] and one study reported 27 % having a history of neuropathy [22]. As diabetics frequently suffer from neuropathy and report mixed sensations such as burning, stinging, pain, this may explain why itch is not the dominating symptom and was less prevalent in our cohort. Due to small nerve damage in long-term diabetes, patients may experience reduced sensations of different nerve stimuli including itch.

Prior investigations suggest optimal dialysis efficacy in terms of higher Kt/V and better nutrition leading to a reduction of uremic itch [23]. It is necessary to emphasize, however, that the Kt/V of patients in this study was increased from mean 1.05 to 1.24, a baseline Kt/V that is considered insufficient according to the valid European Best Practice Guidelines. Our results confirm previous research reporting no difference in the quantity of dialysis expressed by Kt/V between patients with and without itch, but these studies did not specifically investigate CI according to the international classification [21, 24]. In contrast, others identified higher Kt/V in patients with itch [7]. The elevated levels of parathormone (PTH) and phosphorus in patients with CI of our study partly confirm previous studies but serum calcium was also higher in these studies [3, 5, 7]. While phosphorus levels are instable and influenced by diet, phosphate binders, vitamin D supplementation, and dialysis, creatinine in part reflects muscle mass tending to be higher in younger patients. This is of special interest because in our cohort younger HD patients were more frequently affected by CI [1]. Elevated PTH on the other hand has been suspected to be involved into the pathogenesis of CI in HD patients mainly due to the observation that patients suffering from distinct hyperparathyreoidism with itch have experienced complete relief of itch after parathyreoidectomy but PTH never proved a pruritic potency and the role of PTH in the pathogenesis of CI in dialysis patients is still a matter of debate [18, 25, 26]. Our study is in line with others who did not detect any significant differences between HD patients with and without itch regarding laboratory parameters such as serum calcium, sodium, potassium, albumin [4, 21]. CRP was not increased in patients with CI compared with patients without CI which is in contrast to observations in small previous studies [27, 28]. As the number of HD patients investigated in GEHIS is representative and the largest that ever determined CRP, the role of CRP may have been overestimated in the pathogenesis of CI in hemodialysis in the past. In summary, the significant difference concerning PTH, creatinine and phosphorus are unlikely to explain why 25 % of HD patients still suffer from CI.

So far, there is no systematic study investigating the role of dialyzer membranes and the development of itch, especially chronic itch. A smaller study observed that patients dialyzed with polysulfone membranes suffered significantly more frequent from CI than those on hemophane and cuprophane membranes 11]. Others did not find any correlation between itch and the type of dialysis membrane used 7]. We identified significantly reduced CI in patients treated with a polysulfone dialyzer (lifetime prevalence) and significantly more frequent CI when they were dialyzed with polyarylethersulfone membranes (all prevalence estimates). Unfortunately we could not obtain any information in these HD patients about previous membrane material used for HD and on the percentage of patients being switched to other filter materials because they complained about itch. One may explain this discrepancy with the fact that meanwhile quality and efficiency of disposables have continuously been improved and therefore findings of older studies have to be transferred with great caution. As previously reported the use of synthetic and high-flux dialyzer membranes significantly increased and this may indicate an effect on the prevalence of CI [3, 5, 9]. Since we did not assess the consecutive volume used within the process of hemodiafiltration, this also remains to be investigated. These aspects influence the impact of the results on dialyses membranes which have to be interpreted with caution but should definitely encourage future research on this aspect of CI in HD patients.

Heparin respectively the type of heparin used during HD treatment has not been reported to be relevant for CI in HD patients but interestingly, heparin treatment was suggested for treatment of uremic itching [29, 30]. We are not aware of any study that ever investigated heparin use in a large cohort of HD patients like we did now. It must be considered that, just as with the membrane material used, the type of heparin may have been changed once a patient complained about itch but this was not documented by any hemodialysis unit. Moreover, the number of patients treated with low molecular heparin was very small compared to the ones who received unfractionated heparin but nevertheless this is a very interesting observation that needs further investigation.

## Conclusions

As previously discussed some limitations of the study need to be considered and for the current analyses the following ones need to be mentioned [1]. We were not able to include every HD patient in large dialysis units because patient recruiting had to be finished within each dialysis unit after reaching the sufficient number of patients according to the study design (cluster sampling). Critically or acutely ill dialysis patients were not included in this study because acute dialysis units within emergency departments were excluded. This aspect is an ancillary limitation because we aimed to investigate the prevalence of CI in HD patients and not itch in acute renal insufficiency. As data were obtained by self-reporting, a bias due to social desirability is possible. A potential information bias may also apply to variables analyzed in this study due to patients’ misclassification of the outcome variable. HD patients take many different drugs, some may influence lab values e.g. PTH, and some may be considered as a potential trigger of CI [31]. Besides, some patients may have taken anti-itch medication. As our sample is representative and chronic itch (CI) (not acute itch) was addressed, we believe that these aspects can be less considered. Our study adds new findings in the field of epidemiological studies on CI in hemodialysis. Due to the comparability concerning standards in quality of dialysis in developed countries, our study results can be transferred to other Western countries. The first findings of GEHIS indicate CI to occur more frequently in HD patients of younger age, having an etiology of ESRD of primary chronic glomerulonephritis and a history of eczema and dry skin [1]. New analyses show that diabetes is associated with a lower prevalence of CI. We found evidence that the time on HD is associated with a higher prevalence of CI as well as the type of dialyzer membranes used. The elevated levels of PTH, phosphorus and creatinine may represent an epiphenomenon only associated with CI but related to the pathophysiological situation in hemodialysis. All this contributes to the assumption that the pathogenesis of CI in hemodialysis is considered as unclear [18] and we can now add that chronic itch in hemodialysis is probably of multifactorial origin. It is most likely that several factors e.g. PTH elevation, origin of ESRD, type of dialysis membrane, type of heparin used for anticoagulation and a neuropathic component all contribute to the occurrence of CI in HD. All these possible pathogenic factors need to be investigated in future studies. As we could show in another study that the 12-month incidence of CI equals 7 % even in the general population, one has to face that there is a constant number of new itch cases in HD which will without much doubt be higher than in the general population [32].

## Abbreviations

CI: Chronic itch
DOPPS: dialysis outcome and practice patterns study
ESRD: end-stage renal disease
GEHIS: German epidemiological hemodialysis itch study
HD: hemodialysis
HRQOL: health-related quality of life
Kt/V: number used to quantify hemodialysis and peritoneal dialysis treatment adequacy
PTH: parathormone
SD: standard deviation
VAS: visual analogue scale
